# The effect of diurnal distribution of carbohydrates and fat on glycaemic control in humans: a randomized controlled trial

**DOI:** 10.1038/srep44170

**Published:** 2017-03-08

**Authors:** Katharina Kessler, Silke Hornemann, Klaus J. Petzke, Margrit Kemper, Achim Kramer, Andreas F. H. Pfeiffer, Olga Pivovarova, Natalia Rudovich

**Affiliations:** 1 Dept. of Clinical Nutrition, German Institute of Human Nutrition Potsdam-Rehbruecke, 14558 Nuthetal, Germany; 2 German Center for Diabetes Research (DZD), 85764 München-Neuherberg, Germany; 3 Dept. of Endocrinology, Diabetes and Nutrition, Campus Benjamin Franklin, Charité University of Medicine, 12203 Berlin, Germany; 4 Research Group Physiology of Energy Metabolism, German Institute of Human Nutrition Potsdam-Rehbruecke, 14558 Nuthetal, Germany; 5 Laboratory of Chronobiology, Institute for Medical Immunology, Charité University of Medicine, 10117 Berlin, Germany; 6 Division of Endocrinology and Diabetes, Department of Internal Medicine, Spital Bülach, 8180 Bülach, Switzerland

## Abstract

Diurnal carbohydrate and fat distribution modulates glycaemic control in rodents. In humans, the optimal timing of both macronutrients and its effects on glycaemic control after prolonged consumption are not studied in detail. In this cross-over trial, 29 non-obese men were randomized to two four-week diets: (1) carbohydrate-rich meals until 13.30 and fat-rich meals between 16.30 and 22.00 (HC/HF) *versus* (2) inverse sequence of meals (HF/HC). After each trial period two meal tolerance tests were performed, at 09.00 and 15.40, respectively, according to the previous intervention. On the HF/HC diet, whole-day glucose level was increased by 7.9% (p = 0.026) in subjects with impaired fasting glucose and/or impaired glucose tolerance (IFG/IGT, n = 11), and GLP-1 by 10.2% (p = 0.041) in normal glucose-tolerant subjects (NGT, n = 18). Diet effects on fasting GLP-1 (p = 0.009) and PYY (p = 0.034) levels were observed in IFG/IGT, but not in NGT. Afternoon decline of glucose tolerance was more pronounced in IFG/IGT and associated with a stronger decrease of postprandial GLP-1 and PYY levels, but not with changes of cortisol rhythm. In conclusion, the HF/HC diet shows an unfavourable effect on glycaemic control in IFG/IGT, but not in NGT subjects. Consequently, large, carbohydrate-rich dinners should be avoided, primarily by subjects with impaired glucose metabolism.

The control of diurnal glucose fluctuations is a crucial component of body homeostasis. Nutritional approaches are corner stones to achieving euglycaemia in diabetes and health. Today, the best dietary strategy remains unclear. The benefits of wholegrain, fruits, vegetables, nuts and legumes have been widely recognized^1^. However, recently published studies suggest that meal timing and daily eating patterns help prevent metabolic diseases^2^,^3^, pointing towards a pivotal role of circadian rhythms in metabolism^4^.

Circadian rhythms are self-sustained ~24 h rhythms in behaviour, physiology and metabolism that allow organisms the adaptation to the daily recurring day-night cycles and resultant changes in food availability^2^. Consequently, numerous processes of glucose, cholesterol and lipid metabolism, detoxification pathways and immune responses^5^ display circadian oscillation.

Recent studies in rodents provide evidence that the timing of macronutrient consumption influences the circadian clock machinery and metabolism^6^,^7^,^8^,^9^,^10^. Under an ad libitum high fat diet, mice consume an abnormally high proportion of calories during the light phase (i.e. the sleep phase for nocturnal animals) which leads to increased adiposity and decreased glucose tolerance^8^. Remarkably, when the high fat diet was restricted to the active phase of day, mice were protected against obesity, hyperinsulinemia, hepatic steatosis and inflammation^9^. Similar results have been reported for liquid sugar and fructose intake^11^,^12^.

These observations support the hypothesis that consumption of fat and carbohydrates at certain time windows within the active phase might beneficially modulate metabolic homeostasis^13^. Indeed, in mice a low-fat carbohydrate-rich diet at the end of the active phase led to reduced body weight and improved glucose tolerance; conversely, mice on a high fat diet at the end of the active phase showed glucose intolerance, adiposity and features of the metabolic syndrome^14^,^15^. Intervention studies by Sofer *et al*. confirm the beneficial effect of carbohydrate consumption in the evening (end of the active phase)^16^,^17^. Eating carbohydrates mostly at dinner within a hypocaloric diet led to more pronounced weight loss, reduced hunger scores and improved metabolic status in obese subjects^16^. Similarly, carbohydrate consumption at end of the active phase resulted in the improved feeding regulation and amelioration of inflammatory parameters in mice^17^. An improvement of glycaemic control was also observed when carbohydrates were mainly eaten at dinner and protein mainly at lunch^18^. In contrast, epidemiological studies propose a beneficial effect of a carbohydrate-rich diet at the beginning of the day: an increasing carbohydrate intake at the expense of fat in the morning was shown to be protective against the development of diabetes^19^ and metabolic syndrome^20^.

Interestingly, a range of studies showed that identical meals result in higher postprandial elevation of plasma glucose in the evening than in the morning in healthy subjects suggesting a diurnal variation of glucose tolerance during the investigation day^21^,^22^,^23^. In particular, Morgan *et al*. compared the metabolic effects of varying both dietary glycaemic index (GI) and the time at which most daily energy intake was consumed^24^. Consumption of an energy-rich meal in the evening led to significantly higher glucose and insulin response compared to its consumption in the morning. Markedly, this effect was most pronounced in the evening on a high GI diet, confirming that the quality of carbohydrates at a particular time of the day acutely influences glycaemic control throughout the day^24^,^25^.

Taken together, these studies suggest that time of day-dependent carbohydrate and fat intake alters metabolism both in rodents and humans. Thus the detection of the best time for carbohydrate (and fat) consumption in humans as well as the effect of a prolonged consumption of such diet for glycaemic control are of scientific and clinical interest. We therefore investigated the metabolic effect of four-week HC/HF diet (carbohydrate-rich meals in the morning and fat-rich meals in the afternoon) *versus* HF/HC diet (the inverse sequence of meals) in male subjects without diabetes. The primary objective of our study was to compare the effect of both diets on fasting and whole-day levels of glucose and glucose-regulating hormones. Our secondary objective was to analyse the effect of daily carbohydrate and fat distribution on diurnal variation in glucose tolerance. The main findings of this study are: firstly, the HF/HC diet shows an unfavourable effect on the glycaemic control in subjects with an impaired glucose tolerance, but not in subjects with a normal glucose tolerance; secondly, this effect could be explained by the stronger afternoon decline of glucose tolerance in subjects with impaired glucose metabolism.

## Results

### Study subjects, dietary compliance, and anthropometric parameters

**Figure 1:**
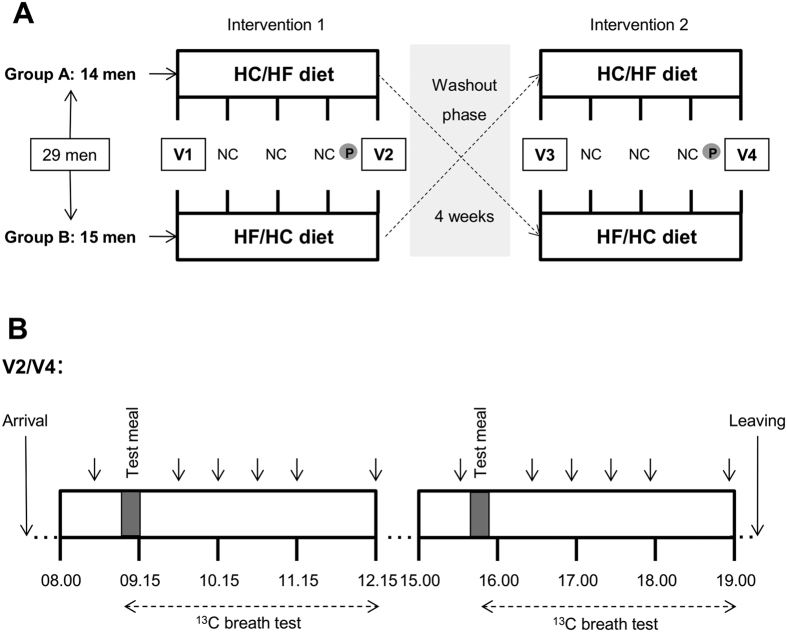
Study design. (**A)** In this cross-over study, two four-week dietary intervention periods were separated by a washout phase. HC/HF diet, isocaloric carbohydrate-rich meals until 13.30 and isocaloric fat-rich meals between 16.30 and 22.00; HF/HC diet, reversed order of meal sequence; V, visit; NC, nutritional counselling; P, pedometer. (**B**) Clinical investigation day. At 09.00 and 15.40 a standardized test meal – fat-rich or carbohydrate-rich – (grey bars) was provided according to participant’s previous intervention. Blood samples (arrows) were drawn and a ^13^C breath test (dotted arrows) performed.

In this cross-over trial, non-obese male subjects without diabetes were randomized to two isocaloric four-week diets: (1) carbohydrate-rich meals until 13.30 h and fat-rich meals between 16.30 h and 22.00 h (HC/HF) *versus* (2) the inverse sequence of meals (HF/HC) (Fig. 1). Within each diet energy intake was equally distributed between the morning and afternoon. Interventions were followed by 12 h investigation days with carbohydrate-rich (MTT-HC) and fat-rich (MTT-HF) meal tolerance tests (MTT), provided either at 09.00 or 15.40, according to the participant’s previous dietary intervention (Fig. 1).

Between January 2014 and July 2015, 32 men started the trial. Three participants dropped out (Supplemental Figure 1). 29 subjects (age 45.9 ± 2.5 years, body mass index (BMI) 27.1 ± 0.8 kg/m^2^) completed the trial. 18 subjects were normal glucose tolerant (NGT); 11 showed an impaired fasting glucose (IFG) and/or an impaired glucose tolerance (IGT) (Table 1). Compared with NGT group, IFG/IGT subjects showed higher fasting triglyceride levels (p = 0.044) and a tendency towards an increased fasting glucose levels (p = 0.064) (Table 1). Chronotype distribution of study subjects is shown in Supplemental Fig. 2A.

Adherence to dietary plans was good, with very similar compliances for the HC/HF diet and the HF/HC diet. For the HC/HF diet, 12073.4 ± 442.3 kJ were consumed in the course of a day, consisting of 49.1 ± 0.7 energy percent (EN%) carbohydrates (CHO), 36.3 ± 0.7 EN% fat and 14.7 ± 0.2 EN% protein. For the HF/HC diet, 11826.3 ± 415.1 kJ were consumed in the course of a day, consisting of 48.7 ± 0.7 EN% CHO, 36.7 ± 0.8 EN% fat and 14.6 ± 0.2 EN% protein. There was no difference between the two diets regarding energy intake, macronutrient composition, amount of saturated fatty acids, fibre and starch as well as GI (Supplemental Table 1). A detailed fragmentation of the compliance for the morning part (06.00–13.30) and the afternoon part (16.30—22.00) is shown in Supplemental Table 1. No major problems consuming the prescribed foods were reported.

Despite of extensive dietary advice, body weight slightly declined after both diets (−0.7% for NGT on the HC/HF diet, non-significant on the HF/HC diet and for IFG/IGT) without difference between the diets (Tables 2 and 3).

### Fasting parameters in response to the HC/HF diet *versus* the HF/HC diet

After four weeks of intervention, both the HC/HF diet and the HF/HC diet reduced fasting levels of glucose, C-peptide, glucagon and lipid parameters (total, HDL and LDL cholesterol) in NGT subjects (Table 2). Similarly, fasting levels of glucose, C-peptide, and HDL cholesterol declined in IFG/IGT subjects (Table 3). For these parameters, no significant differences between the effects of the HC/HF and the HF/HC diet and between NGT and IFG/IGT groups were observed. Interestingly, fasting glucagon like peptide 1 (GLP-1) and peptide YY (PYY) levels showed a diet effect in IFG/IGT subjects (p = 0.009 and p = 0.034, respectively), but not in NGT subjects. Indeed, in the IFG/IGT group, the HC/HF diet induced a strong reduction of GLP-1 level by 45%, whereas no significant change after the HF/HC intervention was found. Fasting PYY also declined after the HC/HF diet by 4.5% and was not altered after the HF/HC diet (Table 3). In the NGT group, post-intervention fasting glucose dependent insulinotropic peptide (GIP) levels were higher after the HC/HF diet relative to the HF/HC diet, but its diet-induced changes did not differ between interventions (Table 2). No effect on insulin sensitivity measured by HOMA-IR was observed in either of the groups (Table 2 and 3).

### Whole-day levels in response to the HC/HF diet *versus* the HF/HC diet

To determine the effect of both diets on whole-day levels, integrated areas under the curve (AUC) over both MTT (AUCday) were calculated as described in Methods. Again, different diet effects in NGT and IFG/IGT subjects were found. AUCday for glucose was increased by 7.9% on the HF/HC diet compared with the HC/HF diet in IFG/IGT subjects, but not in NGT subjects (Table 4). Conversely, a 10.2% increase in whole-day GLP-1 was observed on the HF/HC diet in NGT subjects, but not in IFG/IGT subjects. On the HF/HC diet, AUCday for free fatty acids (FFA) was significantly increased in IFG/IGT subjects compared to NGT subjects (Table 4). Statistical analysis revealed no difference in diet-induced changes between NGT and IFG/IGT groups. Whole-day levels of other studied parameters (Table 4) including hunger and satiety scores (Supplemental Figure 3) showed no differences between the diets and NGT and IFG/IGT subjects.

### Diurnal variation of the metabolic response to MTT-HC

To analyse the effects of both diets on the diurnal variation of glycaemic control, we compared postprandial responses of glucose and glucose-regulating hormones to the same meal (MTT-HC or MTT-HF) in the morning and in the afternoon in the NGT and IFG/IGT group. For this, we defined the diurnal variation in a variable as Δ = afternoon value − morning value.

**Figure 2:**
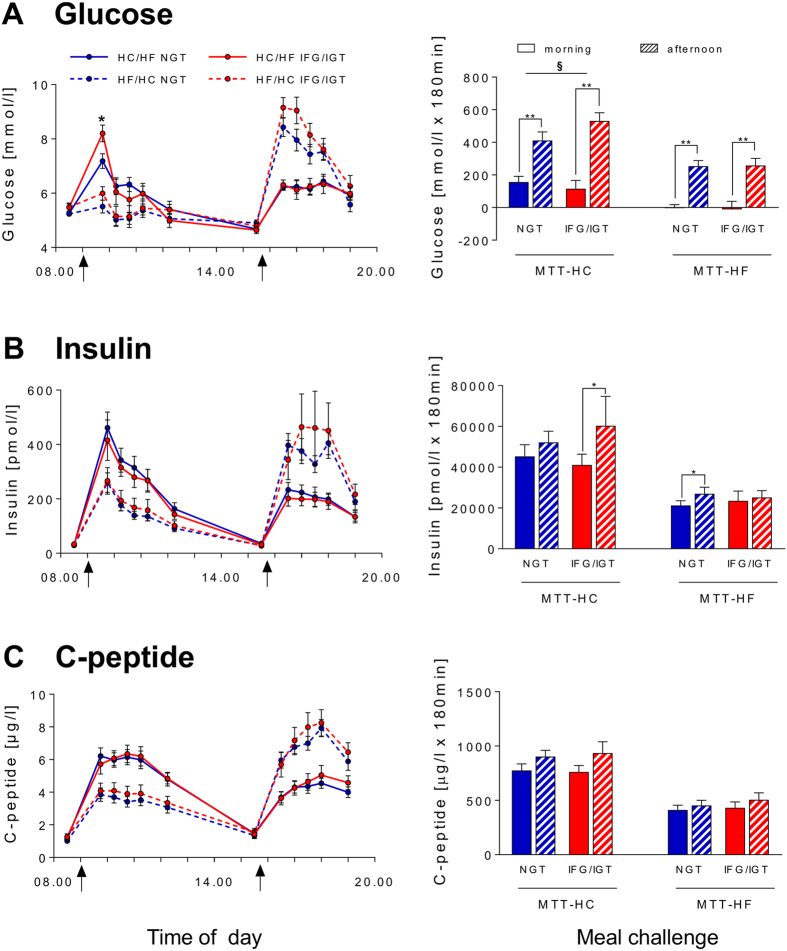
Effects of the HC/HF diet (solid lines) and the HF/HC diet (dotted lines) on pre- and postprandial glucose (**A**), insulin (**B**) and C-peptide (**C**) levels (left panel) and iAUC_0-180_ (right panel) in NGT subjects (blue) and IFG/IGT subjects (red). Here and elsewhere: arrow - test meal; MTT-HC, carbohydrate-rich meal tolerance test; MTT-HF, fat-rich meal tolerance test. Left panel: *p < 0.05 - NGT subjects *vs* IFG/IGT subjects for HC/HF diet; ^#^p < 0.05 – NGT subjects *vs*. IFG/IGT subjects for HF/HC. Right panel: *p < 0.05, **p < 0.01 - afternoon *vs*. morning; ^§^p < 0.05 – NGT subjects *vs*. IFG/IGT subjects for diurnal variation (afternoon value - morning value).

Analysis of the metabolic response to MTT-HC revealed an impairment of glucose tolerance in the afternoon. For glucose, postprandial peak and the incremental area under the curve (iAUC_0-180_) were markedly higher in the afternoon than in the morning. Importantly, afternoon increase of iAUC_0-180_ for glucose was much more pronounced in IFG/IGT subjects compared with NGT subjects (4.5-fold vs. 2.5-fold, respectively) (Fig. 2A). This suggests a stronger decrease of glucose tolerance in IFG/IGT individuals at the end of the day. Notably, after peaking, glucose levels deceased rapidly after the morning meal, whereas in the afternoon glucose levels persisted and remained significantly higher (Fig. 2A).

For insulin, the meal-induced response was rapid and fast in the morning, with peak levels at 30 minutes, while in the afternoon, insulin secretion peaked later (approximately at 2 h). Yet, the overall insulin secretion (iAUC_0-180_) was increased in the afternoon only in IFG/IGT subjects (Fig. 2B). Early and overall indices of insulin secretion (iAUC_ins/glu 0-30_ and iAUC_ins/glu 0-180_) and Gutt index of insulin sensitivity were decreased in the afternoon both in NGT and IFG/IGT subjects without differences between groups (Table 5). C-peptide mirrored insulin levels (Fig. 2C) but showed no significant diurnal variation, neither did hepatic insulin clearance (iHIC) (Table 5).

**Figure 3:**
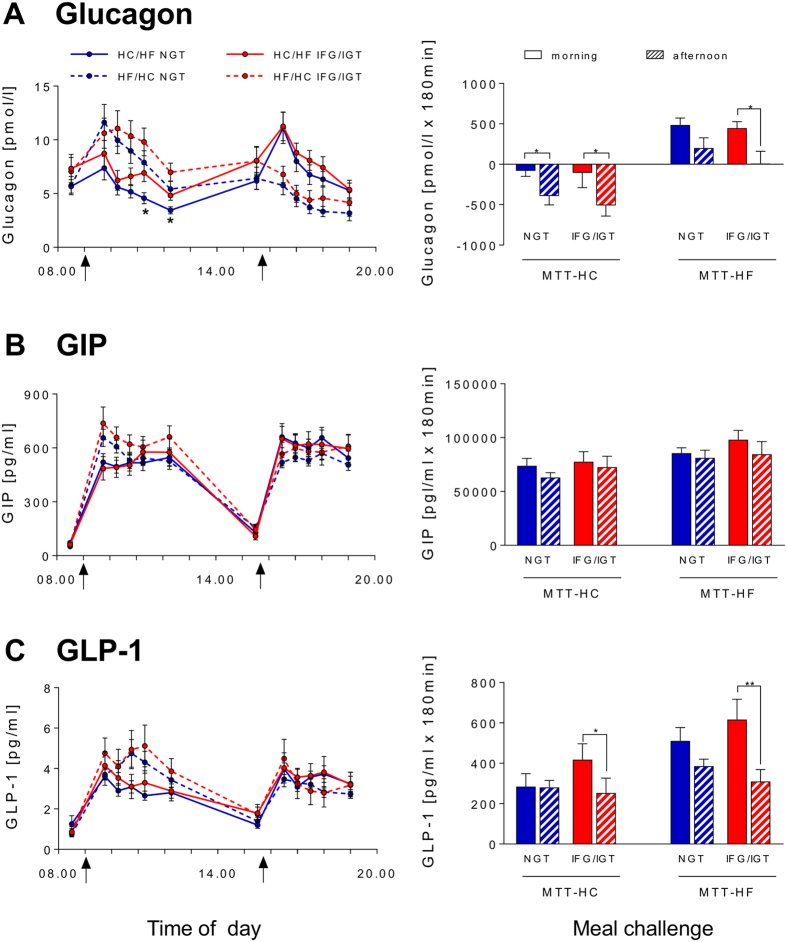
Effects of the HC/HF diet (solid lines) and the HF/HC diet (dotted lines) on pre- and postprandial glucagon (**A**), GIP (**B**) and GLP-1 (**C**) levels (left panel) and iAUC_0-180_ (right panel) in NGT subjects (blue) and IFG/IGT subjects (red).

**Figure 4:**
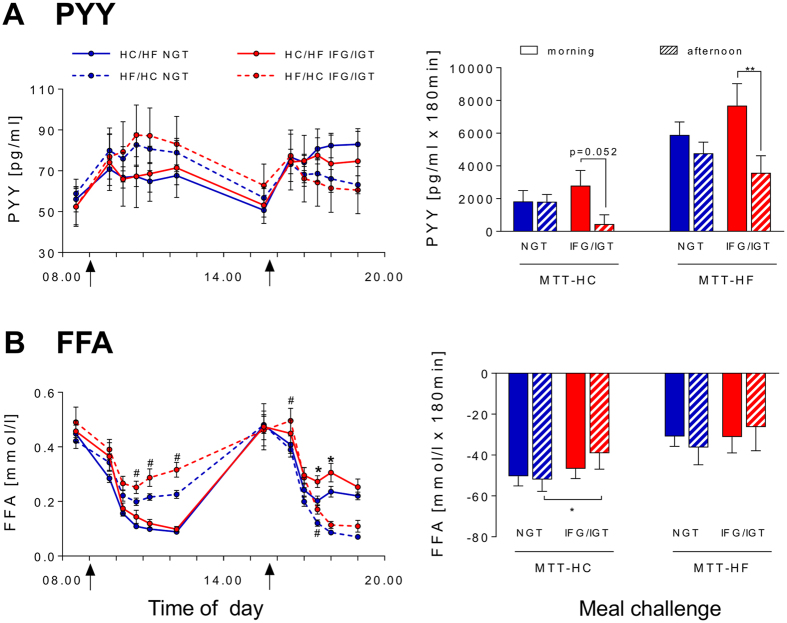
Effects of the HC/HF diet (solid lines) and the HF/HC diet (dotted lines) on pre- and postprandial PYY (**A**) and FFA (**B**) levels (left panel) and iAUC_0-180_ (right panel) in NGT subjects (blue) and IFG/IGT subjects (red).

The iAUC_0-180_ for glucagon was reduced in the afternoon without differences between NGT and IFG/IGT groups (Fig. 3A). Interestingly, GLP-1 secretion showed a pronounced afternoon decline only in IFG/IGT subjects (Fig. 3C) (p = 0.188 for difference of Δ between NGT and IFG/IGT). Similarly, postprandial PYY secretion also showed a trend for diminished afternoon levels only in IFG/IGT subjects (Fig. 4A) (p = 0.071 for difference of Δ between NGT and IFG/IGT). Postprandial GIP decreased in the afternoon, but not significantly (Fig. 3B). FFA response showed no significant diurnal variation (Fig. 4B), although afternoon iAUC_0-180_ for FFA was higher in IFG/IGT subjects (p = 0.044) because of the reduced early postprandial suppression.

Correlation analysis of diurnal differences revealed that, in NGT subjects, diurnal variation of glucose levels (Δ iAUC_0-180_) positively correlated with insulin variation (r = 0.595, p = 0.009). Remarkably, in IFG/IGT subjects, glucose excursion (Δ iAUC_0-180_) did not correlate with insulin, but correlated negatively with diurnal glucagon (r = −0.709, p = 0.015), GLP-1 (r = −0.809, p = 0.003) and PYY (r = −0.845, p = 0.001) variation. Moreover, PYY decline correlated with a decrease in glucagon (r = 0.664, p = 0.026), GIP (r = 0.618, p = 0.043) and GLP-1 (r = 0.845, p = 0.001) response throughout the day.

Analysis of gastric emptying revealed no significant diurnal variation although a trend towards slower gastric emptying in the afternoon was observed (Table 5).

### Diurnal variation of the metabolic response to MTT-HF

As expected, postprandial glucose, insulin, and C-peptide levels were lower and postprandial glucagon, GIP, GLP-1, PYY and FFA levels were higher after the MTT-HF compared with MTT-HC. As for MTT-HC, analysis of the metabolic response to MTT-HF revealed a decrease of glucose tolerance as the day progresses. In the afternoon, peaking of glucose levels was delayed and increased, and iAUC_0-180_ strongly increased in both NGT and IFG/IGT subjects without difference between these groups (Fig. 2A). For insulin, iAUC_0-180_ was significantly higher in the afternoon only in NGT subjects (Fig. 2B). Both insulin and glucose rapidly decreased in the morning and remained elevated in the afternoon (Fig. 2A,B). For C-peptide, peak levels were delayed and increased, although the overall secretion was not dependent on the time of the day (Fig. 2C). In the afternoon, Gutt index showed an afternoon decline in both groups, which was significant only in NGT subjects (Table 5). Diurnal variation of insulin secretion index iAUC_ins/glu_ did not reached statistical significance (Table 5). iHIC decreased in the afternoon only in NGT subjects (Table 5).

Postprandial glucagon decline in the afternoon reached statistical significance only in IFG/IGT subjects (Fig. 3A). Postprandial GIP did also not decrease significantly (Fig. 3B). Similarly to the MTT-HC, GLP-1 and PYY secretion showed pronounced afternoon decline in IFG/IGT subjects (Figs 3C and 4A) (p = 0.188 and p = 0.083, respectively, for difference of Δ between NGT and IFG/IGT). Postprandial FFA levels were higher in IFG/IGT subjects, but no significant diurnal variation was found in either of the groups (Fig. 4B).

Correlation analysis of diurnal differences for MTT-HF revealed that, in NGT subjects, diurnal variation of glucose levels (Δ iAUC_0-180_) correlated with response variation of insulin (r = 0.738, p < 0.001) and glucagon (r = −0.556, p = 0.020); and insulin and glucagon variations were associated with each other (r = −0.568, p = 0.17). In IFG/IGT subjects, glucose variation correlated positively with diurnal insulin pattern (r = 0.745, p = 0.008).

### Diurnal variation of cortisol levels on the HC/HF diet *versus* the HF/HC diet

Because the impairment of the glycaemic control in the evening is related to the cortisol rhythm^26^, we analysed diurnal variation of circulating cortisol level on both diets. As expected, cortisol levels were higher in the morning than in the afternoon (Supplemental Figure 2B). We observed no differences in diurnal cortisol variation (defined as Δ) between diets and between NGT and IFG/IGT subjects (Table 5, Supplemental Figure 2B). Moreover, no correlation of diurnal cortisol variation with afternoon increase of iAUC_0-180_ for glucose and insulin and with afternoon decrease of insulin sensitivity (defined as Gutt index) was found.

## Discussion

The present study is, to our knowledge, the first human trial investigating the effect of a prolonged diurnal distribution of carbohydrate and fat intake on glycaemic control. Surprisingly, we found different metabolic responses in subjects with different stages of glucose tolerance. The HF/HC diet shows an unfavourable effect on the glycaemic control in IFG/IGT subjects, but not in NGT subjects.

Indeed, in IFG/IGT subjects, whole-day glucose level was increased by 7.9% on the HF/HC diet, whereas in NGT subjects it did not differ between the diets. Notably, in NGT group, whole-day level of the incretin hormone GLP-1 was increased by 10.2% on the HF/HC diet, which, however, was not accompanied by an increase in whole-day insulin level. A differential regulation between NGT and IFG/IGT subjects is further proposed by the differences in fasting parameters between the two groups. For NGT, none of the fasting parameters showed a diet effect suggesting a compensatory mechanism in healthy subjects^27^. However, IFG/IGT subjects showed a diet effect for fasting GLP-1 and PYY levels, suggesting that people with an impaired glycaemic control are more susceptible to a diurnal carbohydrate and fat distribution. Remarkably, in neither group, we observed a different effect of the high-carbohydrate and high-fat afternoon meal on fasting triglyceride and fatty acid levels, described previously^28^.

Our data on the unfavourable effect of the HF/HC diet on the glycaemic control in IFG/IGT subjects are in contrast to the results of the mouse study showing that a carbohydrate-rich diet at the end of the active phase led to improved glucose tolerance^14^. Our observation is of great clinical importance suggesting that the avoidance of carbohydrate-rich meals in the evening in people with impaired glucose tolerance should be recommended. In line with this recommendation are previous studies showing that an increasing carbohydrate intake at the expense of fat in the morning seems protective against the development of diabetes^19^ and metabolic syndrome^20^. Conversely, consumption of high GI food in the evening seems most detrimental for human health^24^.

To understand the mechanisms of the unfavourable effect of the HF/HC diet, we compared the effects of both diets on diurnal variation of glycaemic control. In our study, postprandial glucose responses were largely increased and delayed in the afternoon independent of meal composition, confirming the decline in glucose tolerance in the evening, as demonstrated in healthy humans by a range of previous studies^21^,^22^,^23^,^29^. Importantly, the evening decline of glucose tolerance was more pronounced in IFG/IGT subjects. We observed this phenomenon only for the high-carbohydrate meal, which could be explained by the larger postprandial glucose excursion that allowed detecting diurnal differences between morning and afternoon meal. Similar results were shown in an earlier study examining oral glucose tolerance tests in large cohorts in the morning (0930 h) and early afternoon (1300–1400 h)^30^. In this study, subjects with normal fasting glucose showed a small diurnal variation in their glucose tolerance, with less homeostatic control in their afternoon test, while subjects with mildly elevated fasting glucose showed an enhanced diurnal variation^30^. Recent studies confirm the progressive increase in 24 h glycaemic variability from NGT to IFG/IGT subjects^31^,^32^. Sonnier *et al*. showed that insulin resistant subjects suffered from a loss of rhythm in insulin sensitivity, which was partially compensated by an enhancement of the rhythm in insulin levels^26^. Two other studies found increased 24 h profiles of glucose and C-peptide in IFG subjects^33^ and first-degree relatives of type 2 diabetic patients^34^, with conflicting results regarding 24 h insulin levels.

The higher postprandial glucose response to the high-carbohydrate meal in combination with the worsened glucose tolerance in IFG/IGT subjects in the afternoon might explain the increase in whole-day glucose level, observed on the HF/HC diet in IFG/IGT subjects. These results are of great practical importance as they extend the current evidence on the favourable effect of high caloric intake in the morning over high caloric intake in the evening^35^,^36^. Eating the main meal early in the day may therefore be a beneficial strategy to counteract the afternoon/evening impairment of glycaemic control.

Multiple mechanisms contribute to the reduced glucose tolerance in the evening^13^,^37^ including decreased insulin sensitivity, elevated hepatic glucose production, and decreased β cell function. In healthy humans, both insulin sensitivity and β cell responsivity to glucose are reduced in the evening^38^ whereas the data on the diurnal hepatic glucose production are controversial^22^,^39^,^40^. Consistent with previous observations^22^,^41^, we found an increase in insulin resistance and a decrease in early insulin secretion in the afternoon.

Experiments with tissue-specific disruption of circadian rhythms in rodents have identified major roles of peripheral clocks in glucose homeostasis^42^,^43^. Protocols precisely controlling behavioural rhythms showed that the endogenous circadian time is a powerful determinant of glycaemic control in humans^23^. Multiple other factors modulate the rhythm of glucose tolerance in humans including sleep/wake cycle and daily variation of cortisol^44^, adrenocorticotropic hormone^45^, glucagon^22^ and incretin secretion^21^, gastric emptying, as well as fatty acid metabolism^29^.

In our study, the worsened glucose tolerance in IFG/IGT subjects in the afternoon was associated with a stronger decrease of postprandial GLP-1 and PYY secretion. Interestingly, for the high-carbohydrate meal, diurnal glucose variation did not correlate with insulin secretion changes, but correlated with declines in glucagon, GLP-1 and PYY secretion in the afternoon. Further, postprandial insulin levels were strongly increased in the afternoon in IFG/IGT subjects, and the diurnal insulin variation was not associated with pattern of glucagon, GLP-1 and PYY secretion. Notably, in NGT subjects this effect was not found, and glucose pattern correlated well with diurnal changes of insulin level. This suggests that in subjects with impaired glucose metabolism the decreased incretin response in the afternoon reinforced insufficient insulin secretion with consequences of higher postprandial glycaemic levels.

GLP-1 has previously been identified as a potent modulator of the diurnal variation of glycaemic control: Lindgren *et al*. show that early postprandial secretion of both incretin hormones, GIP and GLP-1, is increased in the morning compared to the evening, suggesting that the diurnal rhythm in GIP and GLP-1 levels might be one of potentially many mechanisms accounting for the increased insulin sensitivity in the morning^21^. The mechanism of the higher incretin responses in the morning is not known, and might be associated with diurnal variation of gastric emptying rate^46^ or vagal tone. In our study, we did not find a diurnal variation of gastric emptying rate although a trend towards slower gastric emptying in the afternoon was observed for the MTT-HC. Notably, we observed a marked afternoon decline of GLP-1 secretion (more pronounced in IFG/IGT subjects), but only moderate diurnal variation of GIP secretion. There has been a long-standing debate whether reduced GLP-1 levels are a “universal characteristic” of IFG/IGT and diabetic subjects^47^. Our data indicate that diurnal variation of GLP-1 levels might be modulated by the stages of glucose tolerance.

As GLP-1, PYY displays diurnal variation peaking at 1500 h and subsequently decreasing until the early morning^48^. Recent studies suggest that the stage of glucose tolerance affects PYY secretion with diminished postprandial secretion in diabetic subjects^49^. As for GLP-1, our data propose that particularly the diurnal variation of PYY levels might be affected by the stage of glucose tolerance. Interestingly, a study in primates also suggests a diurnal variation of PYY effects: infusion of PYY reduced the initial rate of eating only during the morning meal, but not during the evening meal^50^. This phenomenon might explain why we did not detect a difference in hunger and satiety scores between NGT and IFG/IGT subjects in our study.

Further, our study revealed increased whole-day FFA levels on the HF/HC diet in IFG/IGT subjects compared to the NGT subjects. Moreover, in the afternoon, iAUC_0-180_ was higher in IFG/IGT subjects after the high-carbohydrate meal than in NGT subjects. Elevated FFA levels are important players in the development of insulin resistance^51^ and diurnal variation of FFA response have been shown to contribute to the decline in glucose tolerance as the day progresses^29^. However, some studies demonstrated no difference in daytime variations between NGT subjects and individuals with IGT^33^,^34^ and therefore this question requires further research.

The next possible mechanism which might contribute in the difference of diet effect in NGT and IFG/IGT subjects is the alteration of cortisol rhythm. As a recent study found that weak cortisol rhythms are associated with greater evening declines in glucose tolerance in prediabetic subjects^26^, we further investigated the possible contribution of this mechanism to the difference in diet effect in NGT and IFG/IGT subjects. Hydrocortisone infused at 1 pm (elevated at abnormal time) versus 5 am (elevated at normal time) results in increased plasma glucose and insulin levels^52^. However, our study observed no differences in diurnal cortisol variation between diets and between NGT and IFG/IGT subjects. Moreover, no correlation of diurnal cortisol variation with evening increase of glucose and insulin levels and decrease of insulin sensitivity was found. Thus, our study could not confirm that the cortisol rhythms contribute to the differences in the diurnal variation in glucose tolerance between NGT and IFG/IGT subjects as described previously^26^.

Finally, strengths and limitations of the current study should be mentioned. The current study has the strength of being a randomized controlled trial with well-defined participants, which is only possible in small-scale studies. Secondly, the duration of our dietary interventions (i.e. four weeks) should be highlighted, as previous reports on a diurnal carbohydrate and/or fat distribution focused on the effect of short-term interventions. Finally, in the current study the macronutrient composition was altered without changing energy intake, by distributing the calories equally between the eating occasions (morning *versus* afternoon part). However, some limitations need to be addressed. Firstly, in spite of extensive nutritional counselling and thoughtful designing of dietary plans, both diets led to a minor weight loss. Possibly, the weight reduction was a reflection of the fact that the participants did not report their true intake in the weighed food records, which is a common problem in human studies^53^. However, the cross-over design of the study should minimize any potential effect of the weight reduction. Secondly, although the overall composition of both diets were very similar and did not show differences in macronutrient composition, fibre and starch content as well as GI, there were minor differences between the fat-rich and carbohydrate-rich diet in the morning versus evening, and we cannot completely exclude that this may influence the results of our study. Thirdly, we cannot completely exclude a contribution of the second-meal phenomenon to the regulation of meal-induced insulin and incretin response in the afternoon^54^. However, the time between MTT1 and MTT2 was long enough (~7 h) to minimize this effect. Finally, our study was conducted in overweight healthy individuals and we cannot exclude that our diets would induce other effects on the glycaemic control in people with obesity and type 2 diabetes. In obese people, it was reported that diurnal variation in glycaemic control was absent^55^. In diabetic patients, the 24 h profile of plasma glucose is impaired and characterized by hyperglycemia in the morning (dawn phenomenon) which is particularly caused by an elevated hepatic glucose production and corresponding increased early morning insulin requirements^56^. In other studies, a loss of diurnal variation after oral glucose administration was described^30^ as well as a time of day-dependence with the highest tolerance at lunch time^57^. Thus, in diabetic patients, results are controversial^30^,^57^ and require further research.

In conclusion, the present study reveals an unfavourable effect of the HF/HC diet on glycaemic control in IFG/IGT subjects, but not in NGT subjects. Consequently, considering the impairment of glucose tolerance as the day progresses, large, carbohydrate-rich dinners may potentially need to be avoided, primarily by individuals with an impaired glycaemic control.

## Methods

### Ethics statement

The study protocol and informed consent document were approved by the Medical Ethics Committee of Charité University Medicine, Berlin, Germany (EA2/074/12) and were in accordance with the Helsinki Declaration of 1975. All subjects gave written informed consent.

The study was registered in May 2015 at clinicaltrial.gov as NCT02487576.

### Study participants

Study participants (18–68 years old) were recruited from Berlin-Brandenburg, Germany. The screening examination of participants included anthropometric measurements, blood sampling, an oral glucose tolerance test (OGTT), indirect calorimetry (CareFusion; Yorba Linda, USA), and interviews on lifestyle and medical history. Men with a BMI between 22 and 34.9 kg/m^2^, fasting venous glucose levels <126 mg/dL and 2 h glucose levels <200 mg/dL in the 75-g OGTT were eligible for participation. Exclusion criteria were weight changes >2 kg within past 2 months, current shift work or history of shift work and diseases or conditions that might influence the outcome of the study.

### Study design

The cross-over study included two four-week dietary intervention periods separated by a washout phase of 31 ± 2 days (Fig. 1). Details on the randomization are stated in Supplemental Methods. The HC/HF diet consisted of isocaloric carbohydrate-rich breakfast and lunch (65 EN% CHO, 20 EN% fat, 15 EN% protein) and fat-rich snack and dinner (35 EN% CHO, 50 EN% fat, 15 EN% protein). The HF/HC diet consisted of the reversed order of meal composition.

### Dietary interventions

For each participant, weighed food records for five consecutive days were analysed with PRODI 6.1 expert software (Nutriscience, Stuttgart, Germany) to determine food preferences and mean caloric intake. To ensure a good compliance, individual dietary plans were formulated for each participant meeting the macronutrient composition of both diets and considering the individual food preferences (Supplemental Tables 2 and 3). As well as possible, dietary plans were controlled for the amount of saturated fatty acids, starch and fibre as well as GI. Dietary plans were isocaloric with the product of the individual resting metabolic rate and the physical activity level and adjusted to the mean daily intake of the food record. Calories of dietary plans were evenly distributed between morning (breakfast + lunch) and afternoon (snack + dinner) leading to a daily macronutrient composition of 50 EN% CHO, 35 EN% fat (14 EN% SFA) and 15 EN% protein.

Participants were instructed to eat breakfast and lunch by 13.30 and snack and dinner 16.30–22.00. Cut-off points were chosen according to usual eating times in Germany to allow a best possible compliance. During the run-in, intervention, and washout periods, participants were asked to avoid alcohol, maintain their normal coffee consumption and follow their regular routine of wakefulness and sleep and physical activity levels. Munich Chronotype Questionnaire (MCTQ)^58^ was used to determine participants’ chronotypes, by determination of the mid-sleep time point on free days adjusted for individual average sleep need accumulated on work days (MSF-Sc). Activity levels were assessed at the end of each intervention using a pedometer (AS 50; Beurer Inc, Ulm, Germany)

### Patient examinations

At all visits, anthropometrical measurements were performed after an overnight fast. Fasted blood samples were drawn from the forearm vein, centrifuged at 1800 g for 10 minutes 4 °C and stored at −80 °C until analysis.

At V2 and V4, two MTTs were performed in the course of the day, i.e. at 09.00 and at 15.40 (Fig. 1B, Supplemental Table 4). MTT-HC was rich in carbohydrates (64.8 EN% CHO, 20.3 EN% fat, 14.8 EN% protein) and contained 835 kcal; MTT-HF was rich in fat (35.3 EN% CHO, 49.6 EN% fat, 15.1 EN% protein) and contained 849 kcal. The chronological order of the MTTs depended on the participant’s previous dietary intervention. Participants ingested the meals within 15 minutes. Blood samples were taken before (−5 minutes) and 30, 60, 90, 120 and 180 minutes after completion of each meal. A ^13^C-acetate breath test was performed along with each MTT to determine gastric emptying rate (Supplemental Methods). Satiety and hunger scores were assessed before (−5 minutes), immediately after (0 minutes) and 180 minutes after each meal as described before^59^. Cortisol levels were determined before and 180 minutes after each meal.

### Sample analyses

Routine laboratory markers were measured using standard methods (ABX Pentra 400; HORIBA, ABX SAS, France). Commercial ELISA were used for measurement of insulin, C-peptide, glucagon (Mercodia, Sweden), GIP, total PYY (Merck Chemicals GmbH, Germany) and cortisol (IBL Internation, Germany) in serum; active GLP-1 was measured by Meso Scale Discovery assay (USA). For measurement of glucagon, GIP and GLP-1, EDTA plasma with 100 μM DPPIV inhibitor and 500 KIU/ml aprotinin were used.

### Sample size and power calculation

Power calculation was completed using the nQuery Advisor 6.0. For the paired parametric design and the sample size of 28 subjects, the current study provided 80% power to detect 5% difference between groups, if the effect size was 0.55. To allow discontinuation, 32 participants started the trial.

### Calculations and statistical analyses

Dietary GI was calculated as described previously^60^. GI values of foods consumed were obtained from the DioGenes database^60^.

Statistical analyses were performed with SPSS v.20 (SPSS, Chicago, IL). To estimate the effects of dietary treatments on anthropometrical and fasting metabolic parameters, changes from baseline (week 4 – week 0, in percent to baseline) were calculated. They were used as dependent variables in a linear mixed-effects model with treatment (HC/HF or HF/HC diets), period (first or second) and residual effect of the first experimental period over the second period as fixed factors, and subjects included as a random factor^61^. In an additional analysis, weight change from baseline was included in the linear mixed model as a covariate. Sampling distribution was analyzed using Shapiro-Wilk test. Not normally distributed data were log-transformed before analysis. The same model was used for parameter levels at the start (week 0) and at the end (week 4) of intervention. Because no effect of the period and no residual effect were observed for any measured variable, for following analyses data were pooled in HC/HF and HF/HC groups.

For FFA, glucose and meal-induced hormone secretion, areas under the curve (AUC) and incremental AUC (after subtraction of the baseline area, iAUC) were determined by trapezoidal method. Integrated AUC of both meal tolerance tests (AUCday) were calculated for analysis of whole-day levels. To compare MTTs in the morning and in the afternoon, iAUC were calculated for the total (0–180 min) responses. For fasting whole-day levels, differences between NGT and IFG/IGT subjects were calculated as the delta percentage (Δ% = (new value − basal value)/basal value * 100). For the comparison between morning and afternoon meal, diurnal variation in a variable was calculated as Δ = afternoon value – morning value.

Insulin secretion was assessed as the ratio of iAUC for insulin to iAUC for glucose (iAUC_ins/glu_). Insulin sensitivity in MTT was determined by the Gutt index (ISI Gutt_0-120_)^62^. Hepatic insulin clearance (HIC) was calculated as ratio of iAUC_0-180_ for C-peptide to iAUC_0-180_ for insulin^63^. HOMA-IR was calculated according to following equation. HOMA-IR [mmol * mU * L^2^] = glucose [mmol/L] × insulin [mU/L]/22.5, using fasting values.

For markers of satiety and hunger scores, daily levels were calculated as the average level from six time points of data collection.

Time-of-day and diet effects were estimated using two-way repeated measures ANOVA. Comparisons between two groups at each time point were performed using paired Student’s t-test or Wilcoxon test, and correlations were calculated using Pearson or Spearman tests, depending on sample distribution. P values < 0.05 were considered significant in all analyses. All data are presented as means ± SEMs.

## Additional Information

**How to cite this article:** Kessler, K. *et al*. The effect of diurnal distribution of carbohydrates and fat on glycaemic control in humans: a randomized controlled trial. *Sci. Rep.* **7**, 44170; doi: 10.1038/srep44170 (2017).

**Publisher's note:** Springer Nature remains neutral with regard to jurisdictional claims in published maps and institutional affiliations.

## Supplementary Material

Supplementary Information

## Figures and Tables

**Table 1:** Clinical characteristics of study participants.

	All	NGT subjects	IGT/IFG subjects	p-value*
N (% male)	29 (100)	18 (100)	11 (100)	
Age [years]	45.90 ± 2.54	43.83 ± 3.34	49.27 ± 3.83	0.306
**Anthropometric measurements**				
Chronotype [MSF-Sc]	3.44 ± 0.19	3.42 ± 0.27	3.45 ± 0.18	0.924
Weight [kg]	87.04 ± 2.85	86.06 ± 3.75	88.65 ± 4.52	0.611
BMI [kg/m^2^]	27.07 ± 0.75	27.13 ± 0.95	26.95 ± 1.31	0.911
Waist circumference [cm]	93.55 ± 2.09	92.06 ± 2.37	96.00 ± 3.95	0.396
Waist-to-hip ratio	0.91 ± 0.01	0.90 ± 0.01	0.94 ± 0.03	0.100
**Lipid metabolism**				
Total cholesterol [mmol/l]	5.24 ± 0.18	5.24 ± 0.17	5.25 ± 0.40	0.978
HDL cholesterol [mmol/l]	1.20 ± 0.04	1.24 ± 0.05	1.13 ± 0.07	0.222
LDL cholesterol [mmol/l]	3.48 ± 0.17	3.51 ± 0.17	3.42 ± 0.37	0.841
Triglycerides [mmol/l]	1.25 ± 0.14	1.08 ± 0.13	1.52 ± 0.30	**0**.**044**
NEFA [mmol/l]	0.49 ± 0.03	0.48 ± 0.04	0.50 ± 0.03	0.834
**Glucose metabolism**				
Glucose [mmol/l]	5.83 ± 0.12	5.65 ± 0.14	6.12 ± 0.21	0.064
Insulin [pmol/l]	34.27 ± 5.22	31.23 ± 6.07	39.22 ± 9.70	0.580
HOMA-IR [mmol*mU *l^−2^]	1.55 ± 0.26	1.36 ± 0.28	1.87 ± 0.50	0.363

Data were collected at visit 1 (start of first intervention period). Data are shown as mean ± SEM. MSF-Sc, mid-sleep time point on free days adjusted for individual average sleep need accumulated on work days determined using MCTQ 58; NGT, normal glucose tolerance, IFG, impaired fasting glucose, IGT, impaired glucose tolerance. *Statistical differences between group NGT and IFG/IGT.

**Table 2:** Fasting parameters in response to HC/HF diet and HF/HC diet in NGT subjects.

	HC/HF diet			HF/HC diet			P^a^	P_corr_^b^
	Pre	Post	Δ%	Pre	Post	Δ%		
Weight [kg]	86.0 ± 3.7	85.5 ± 3.7	−0.7*	86.1 ± 3.8	85.8 ± 3.8	−0.3	0.398	
BMI [kg/m^2^]	27.1 ± 0.9	26.9 ± 0.9	−0.8**	27.2 ± 1.0	27.0 ± 1.0	−0.4	0.283	
**Glucose metabolism**								
Glucose [mmol/l]	5.75 ± 0.13	5.26 ± 0.08	−8.4**	5.70 ± 0.12	5.24 ± 0.09	−8.1**	0.692	0.431
Insulin [pmol/l]	32.48 ± 6.18	31.69 ± 3.45	−2.5	33.00 ± 5.51	29.44 ± 4.56	−10.2	0.584	0.560
C-peptide [μg/l]	2.22 ± 0.65	1.13 ± 0.11	−49.0	2.43 ± 0.73	1.01 ± 0.12	−58.3*	0.994	0.914
HOMA-IR [mmol· mU· l^−2^]	1.42 ± 0.29	1.24 ± 0.14	−12.8	1.42 ± 0.25	1.15 ± 0.18	−19.4	0.576	0.567
Glucagon [pmol/l]	6.62 ± 0.81	5.66 ± 0.63	−14.4*	6.79 ± 0.84	5.75 ± 0.85	−15.4*	0.979	0.950
GIP [pg/ml]	67.28 ± 8.89	71.34 ± 8.52	6.0	62.90 ± 6.72	51.52 ± 4.72^#^	−18.1	0.368	0.383
GLP-1 [pg/ml]	1.86 ± 0.64	1.26 ± 0.40	−34.4	1.20 ± 0.28	0.75 ± 0.10	−37.5	0.701	0.632
PYY [pg/ml]	65.77 ± 8.63	56.12 ± 6.20	−14.7	59.36 ± 5.27	58.84 ± 7.09	−0.9	0.754	0.765
**Lipid metabolism**								
Total cholesterol [mmol/l]	5.23 ± 0.15	4.72 ± 0.17	−9.7**	5.21 ± 0.17	4.82 ± 0.21	−7.5**	0.575	0.807
HDL cholesterol [mmol/l]	1.28 ± 0.05	1.10 ± 0.04	−13.8**	1.23 ± 0.05	1.10 ± 0.04	−10.7**	0.324	0.233
LDL cholesterol [mmol/l]	3.53 ± 0.15	3.18 ± 0.16	−9.9**	3.46 ± 0.18	3.20 ± 0.21	−7.4**	0.435	0.589
Triglycerides [mmol/l]	0.95 ± 0.09	0.98 ± 0.09	−3.8	1.13 ± 0.13	1.12 ± 0.11	−0.2	0.851	0.947
NEFA [mmol/l]	0.48 ± 0.04	0.45 ± 0.03	−6.6	0.51 ± 0.04	0.42 ± 0.03	−17.4	0.115	0.139

*p < 0.05, **p < 0.01 for difference from baseline.

^§^Pre-intervention difference between HC/HF diet and HF/HC diet, p < 0.05.

^#^Post-intervention difference between HC/HF diet and HF/HC diet, p < 0.05.

^a^Comparison of changes after HC/HF diet and HF/HC diet in the linear mixed model

^b^Comparison of changes after HC/HF diet and HF/HC diet in the linear mixed model after correction for weight change.

**Table 3:** Fasting parameters in response to HC/HF diet and HF/HC diet in IGT/IFG subjects.

	HC/HF diet			HF/HC diet			P^a^	P_corr_^b^
	Pre	Post	Δ%	Pre	Post	Δ%		
Weight [kg]	88.2 ± 4.6	87.8 ± 4.6	−0.4	88.7 ± 4.6	88.0 ± 4.5	−0.7	0.363	
BMI [kg/m^2^]	26.8 ± 1.3	26.7 ± 1.3	−0.5	27.0 ± 1.3	26.8 ± 1.3	−0.7	0.394	
**Glucose metabolism**								
Glucose [mmol/l]	6.18 ± 0.22	5.48 ± 0.13	−11.4**	6.07 ± 0.16	5.49 ± 0.15	−9.6**	0.655	0.574
Insulin [pmol/l]	42.53 ± 11.02	33.22 ± 5.00	−21.9	45.87 ± 12.97	33.44 ± 6.79	−27.1	0.719	0.804
C-peptide [μg/l]	2.18 ± 0.44	1.25 ± 0.22	−42.6*	2.53 ± 0.65	1.25 ± 0.18	−50.6*	0.601	0. 515
HOMA-IR [mmol· mU· l^−2^]	2.08 ± 0.56	1.38 ± 0.23	−33.8	2.15 ± 0.64	1.40 ± 0.31	−34.7	0.740	0.802
Glucagon [pmol/l]	8.53 ± 0.01	7.33 ± 1.32	−14.0	8.26 ± 0.90	7.06 ± 1.3	−14.7	0.639	0.949
GIP [pg/ml]	62.63 ± 9.24	61.76 ± 5.46	−1.4	74.47 ± 12.12	57.57 ± 8.05	−22.7	0.826	0.995
GLP-1 [pg/ml]	1.55 ± 0.35	0.85 ± 0.17	−45.0*	1.01 ± 0.23^§^	0.87 ± 0.25^#^	−13.3	**0**.**009**	**0**.**007**
PYY [pg/ml]	55.46 ± 9.33	52.45 ± 9.64	−4.5*	48.78 ± 8.65	52.46 ± 8.88	7.5	**0**.**034**	**0**.**029**
**Lipid metabolism**								
Total cholesterol [mmol/l]	5.19 ± 0.38	4.86 ± 0.34	−6.3	5.30 ± 0.40	4.97 ± 0.32	−6.1	0.285	0.061
HDL cholesterol [mmol/l]	1.18 ± 0.06	1.05 ± 0.05	−10.7**	1.17 ± 0.08	1.03 ± 0.05	−11.9*	0.107	**0**.**016**
LDL cholesterol [mmol/l]	3.34 ± 0.34	3.26 ± 0.29	−2.2	3.47 ± 0.36^§^	3.38 ± 0.27	−2.6	0.648	0.385
Triglycerides [mmol/l]	1.49 ± 0.30	1.21 ± 0.25	−18.7	1.45 ± 0.38	1.24 ± 0.24	−14.3	0.787	0.821
NEFA [mmol/l]	0.54 ± 0.05	0.46 ± 0.04	−14.6	0.45 ± 0.04	0.49 ± 0.06	8.0	0.965	0.929

*p < 0.05, **p < 0.01 for difference from baseline.

^§^Pre-intervention difference between HC/HF diet and HF/HC diet, p < 0.05.

^#^Post-intervention difference between HC/HF diet and HF/HC diet, p < 0.05.

^a^Comparison of changes after HC/HF diet and HF/HC diet in the linear mixed model.

^b^Comparison of changes after HC/HF diet and HF/HC diet in the linear mixed model after correction for weight change.

**Table 4:** Whole-day levels of metabolic markers in response to HC/HF diet and HF/HC diet.

	NGT			IFG/IGT		
	HC/HF diet	HF/HC diet	Δ %	HC/HF diet	HF/HC diet	Δ %
Glucose [mmol/l∙6 h]	2199 ± 45	2233 ± 56	1.6	2196 ± 92	2370 ± 87	7.9*
Insulin [pmol/l∙6 h]	84412 ± 9146	83926 ± 8355	−0.6	78105 ± 9176	94762 ± 20283	21.3
C-peptide [μg/l∙6 h]	1696 ± 117	1740 ± 126	2.6	1748 ± 139	1870 ± 189	7.0
Glucagon [pmol/l∙6 h]	2280 ± 239	2282 ± 291	0.1	2673 ± 258	2653 ± 378	−0.7
GIP [pg/ml∙6 h]	190981 ± 13539	185860 ± 8762	−2.7	192525 ± 20800	207118 ± 18330	7.6
GLP-1 [pg/ml∙6 h]	1113 ± 66	1227 ± 77	10.2*	1203 ± 191	1339 ± 240	11.2
PYY [pg/ml 6 h]	25838 ± 1433	26062 ± 1773	0.9	25383 ± 4627	26273 ± 4423	3.5
FFA [mmol/l∙6 h]	80.3 ± 3.6	79.2 ± 3.5	−1.4	94.6 ± 8.0	100.9 ± 8.0^§^	6.7

For FFA, glucose and meal-induced hormone secretion (insulin, C-peptide, glucagon, GIP, GLP-1, PYY), integrated AUCs after both MTT1 and MTT2 (AUC_day_) are shown. *p < 0.05 for a difference between HC/HF diet and HF/HC diet; ^§^p < 0.05 for a difference between NGT and IGT/IFG subjects.

**Table 5:** Indices of glucose metabolism, gastric emptying and cortisol levels in the morning *versus* afternoon in NGT and IFG/IGT subjects.

	Meal	NGT			IFG/IGT			P-value (Δ NGT vs. Δ IFG/IGT)
		Morning	Afternoon	Δ	Morning	Afternoon	Δ	
**Indices of glucose metabolism**								
iAUC_ins/glu 0-30_	HC	305.3 ± 67.6	91.0 ± 24.3	−214.3	149.1 ± 28.9^§^	73.7 ± 13.6	−**75**.4**	0.122
	HF	622.9 ± 270.9	147.4 ± 23.7	−475.5	361.5 ± 115.4	114.0 ± 20.5	−247.5	1.000
iAUC_ins/glu 0-180_	HC	384.2 ± 189.0	117.7 ± 19.4	−**266**.5*	339.5 ± 142.8	114.0 ± 20.6	−225.5	0.982
	HF	184.8 ± 124.5	81.7 ± 58.2	−103.1	−116.2 ± 137.9	110.9 ± 15.2	227.1	0.191
ISI Gutt_0-120_ [mg*l^2^/ mmol*mU*min]	HC	65.5 ± 3.9	53.5 ± 3.3	−**12**.0*	65.7 ± 5.5	49.8 ± 3.5	−**15**.9**	0.549
	HF	88.6 ± 4.7	75.3 ± 3.2	−13.3**	89.0 ± 7.9	78.8 ± 6.5	−10.2	0.511
iHIC [AU]	HC	6.6 ± 0.5	6.3 ± 0.4	−0.3	6.8 ± 0.6	6.3 ± 0.6	−0.5	0.600
	HF	6.7 ± 0.4	5.9 ± 0.4	−**0**.8**	6.8 ± 0.5	6.8 ± 0.4	0.0	0.130
**Gastric emptying**								
T1/2 [min]	HC	157.6 ± 20.4	172.6 ± 17.4	−15.0	162.4 ± 18.0	197.9 ± 33.4	35.5	0.675
	HF	152.8 ± 12.8	142.9 ± 28.4	−9.9	207.7 ± 31.7	154.5 ± 29.2	−53.2	0.577
Tlag [min]	HC	108.4 ± 13.4	139.9 ± 14.9	31.5	106.5 ± 18.4	158.5 ± 25.9	52.0	0.654
	HF	110.8 ± 11.4	118.5 ± 23.2	7.7	155.0 ± 25.2	118.9 ± 24.8	−36.1	0.540
Cortisol^a^ [ng/ml]	HC/HF	91.7 ± 7.8	34.6 ± 2.8	−57.1**	103.9 ± 12.6	36.9 ± 4.2	−67.0**	0.434
	HF/HC	101.0 ± 6.2	35.2 ± 2.7	−65.8**	100.1 ± 9.4	38.2 ± 5.6	−61.9**	0.716

T1/2 - half gastric emptying time; Tlag - time of fastest gastric emptying; iHIC – incremental hepatic insulin clearance; HC – carbohydrate-rich meal; HF – fat-rich meal. Diurnal variation in a variable is defined as Δ = afternoon value – morning value. Negative values of Δ are referred to as the afternoon decline in the given variable. *p < 0.05, **p < 0.01 for difference between morning and afternoon. ^§^Difference between NGT and IFG/IGT subjects in the morning, p < 0.05. ^a^For cortisol, values before the morning meal and after the afternoon meal are shown.
