# Supplementary material for: The effect of diurnal distribution of carbohydrates and fat on glycaemic control in humans: a randomized controlled trial
Source: Sci Rep. 2017 Mar 8;7:44170. doi: 10.1038/srep44170 (PMC5341154; doi:10.1038/srep44170)
Supplement: Supplementary Information [file srep44170-s1.pdf]

Electronic Supplementary Information for

The effect of diurnal distribution of carbohydrates and fat on glycaemic control in humans: a randomized controlled trial

Katharina Kessler[1,2,3], Silke Hornemann[1,2], Klaus J. Petzke[4], Margrit Kemper[1,2,3], Achim Kramer[5], Andreas F. H. Pfeiffer[1,2,3], Olga Pivovarova[1,2,3] *, Natalia Rudovich[1,2,3,6] *

[1]*Dept. of Clinical Nutrition, German Institute of Human Nutrition Potsdam-Rehbruecke, 14558 Nuthetal, Germany;*

[2]*German Center for Diabetes Research (DZD), 85764 München-Neuherberg, Germany;*

[3]*Dept. of Endocrinology, Diabetes and Nutrition, Campus Benjamin Franklin, Charité University of Medicine, 12203 Berlin, Germany;*

[4]*Research Group Physiology of Energy Metabolism, German Institute of Human Nutrition Potsdam-Rehbruecke, 14558 Nuthetal, Germany;*

[5]*Laboratory of Chronobiology, Institute for Medical Immunology, Charité University of Medicine, 10117 Berlin, Germany*

[6]*Division of Endocrinology and Diabetes, Department of Internal Medicine, Spital Bülach, 8180 Bülach, Switzerland;*

*** N. Rudovich and O. Pivovarova contributed equally to the manuscript.**

Table of Content

Randomization and blinding 3

^{13}C-acetate breath test 3

References 4

Figure S1. Flow of participants 5

Figure S2. Circadian parameters of study participants 6

Figure S3. Hunger and satiety scores in response to the HC/HF diet and the HF/HC diet 7

Table S1. Compliance during both dietary interventions 8

Table S2. Example of a dietary plan for the HC/HF diet 9

Table S3. Example of a dietary plan for the HF/HC diet 10

Table S4. Composition of provided test meals 11

Randomization and blinding

Randomization of study participants was done by a statistician who had no role in study design, data collection and analysis, decision to publish, or preparation of the manuscript. Statistician was blinded regarding the allocation groups. Participants were matched regarding age, BMI, fasting and 2-h glucose levels in the OGTT and subsequently randomized. For randomization a random number generator was used to generate a number between 0 and 1. The obtained number was rounded leading to 0 or 1, respectively, each presenting one of the allocation groups. Allocation was disclosed to both the participant and a nutritionist as soon as available, allowing time for preparation of dietary protocols.

^{13}C-acetate breath test

A ^{13}C-acetate breath test was performed along with each meal tolerance test to determine gastric emptying rate[1]. 60 mg sodium acetate-1-13C (99 atom % 13C, Wagner Analysen Technik GmbH, Bremen, Germany) was swallowed with a sip of the provided water at the end of each meal. Breath samples were taken in duplicates at baseline and every 15 min after ^{13}C-acetate administration into 10 mL tubes (Exetainer, Labco, High Wycombe, UK) for 4-h (morning meal tolerance test) and 3-h (afternoon tolerance test), respectively, for analysis of $^{13}CO_2$ enrichments. Breath [$^{13}CO_2$] enrichments were analyzed by isotope-ratio mass spectrometry (BreathMAT, Thermo Scientific Corp., Bremen, Germany). Isotope composition of carbon was expressed in the conventional delta per mill notation [2]. Evaluation of the breath tests were performed as described[3,4]. Percentage of ^{13}C-recovery from [1-13C]-acetate in $^{13}CO_2$ was calculated according to Schoeller [5] based on delta per mill values over baseline and computed endogenous CO_2 production rate using the body surface area according to Haycock [6]. The time plot of pulmonary [$^{13}CO_2$]-excretion (% dose/h) data were used for mathematical curve fitting using nonlinear regression analysis provided by the Microsoft® Excels Solver procedure and following formula: y = atb • e-ct, where (atb) describes the increase in [$^{13}CO_2$] recovery in breath, (e-ct) describes the washout of the [$^{13}CO_2$] from the breath, t is time in hours and a, b, and c are regression-estimated constants.

The following breath test parameters of gastric emptying were computed: half emptying time (T1/2) and time of fastest emptying (Tlag)[4,7,8].

References

1 Braden, B. *et al.* The [13C]acetate breath test accurately reflects gastric emptying of liquids in both liquid and semisolid test meals. *Gastroenterology* **108**, 1048-1055 (1995).

2 Petzke, K. J. & Klaus, S. Reduced postprandial energy expenditure and increased exogenous fat oxidation in young woman after ingestion of test meals with a low protein content. *Nutr Metab (Lond)* **5**, 25, doi:10.1186/1743-7075-5-25 (2008).

3 Ghoos, Y. F. *et al.* Measurement of gastric emptying rate of solids by means of a carbon-labeled octanoic acid breath test. *Gastroenterology* **104**, 1640-1647 (1993).

4 Schadewaldt, P. *et al.* Application of isotope-selective nondispersive infrared spectrometry (IRIS) for evaluation of [13C]octanoic acid gastric-emptying breath tests: comparison with isotope ratio-mass spectrometry (IRMS). *Clin Chem* **43**, 518-522 (1997).

5 Schoeller, D. A., Klein, P. D., Watkins, J. B., Heim, T. & MacLean, W. C., Jr. 13C abundances of nutrients and the effect of variations in 13C isotopic abundances of test meals formulated for 13CO2 breath tests. *The American journal of clinical nutrition* **33**, 2375-2385 (1980).

6 Haycock, G. B., Schwartz, G. J. & Wisotsky, D. H. Geometric method for measuring body surface area: a height-weight formula validated in infants, children, and adults. *The Journal of pediatrics* **93**, 62-66 (1978).

7 Schommartz, B., Ziegler, D. & Schadewaldt, P. Significance of diagnostic parameters in [13C]octanoic acid gastric emptying breath tests. *Isotopes in environmental and health studies* **34**, 135-143 (1998).

8 van Nieuwenhoven, M. A., Wagenmakers, A. J., Senden, J. M., Brouns, F. & Brummer, R. J. Performance of the [13C]-acetate gastric emptying breath test during physical exercise. *European journal of clinical investigation* **29**, 922-928 (1999).

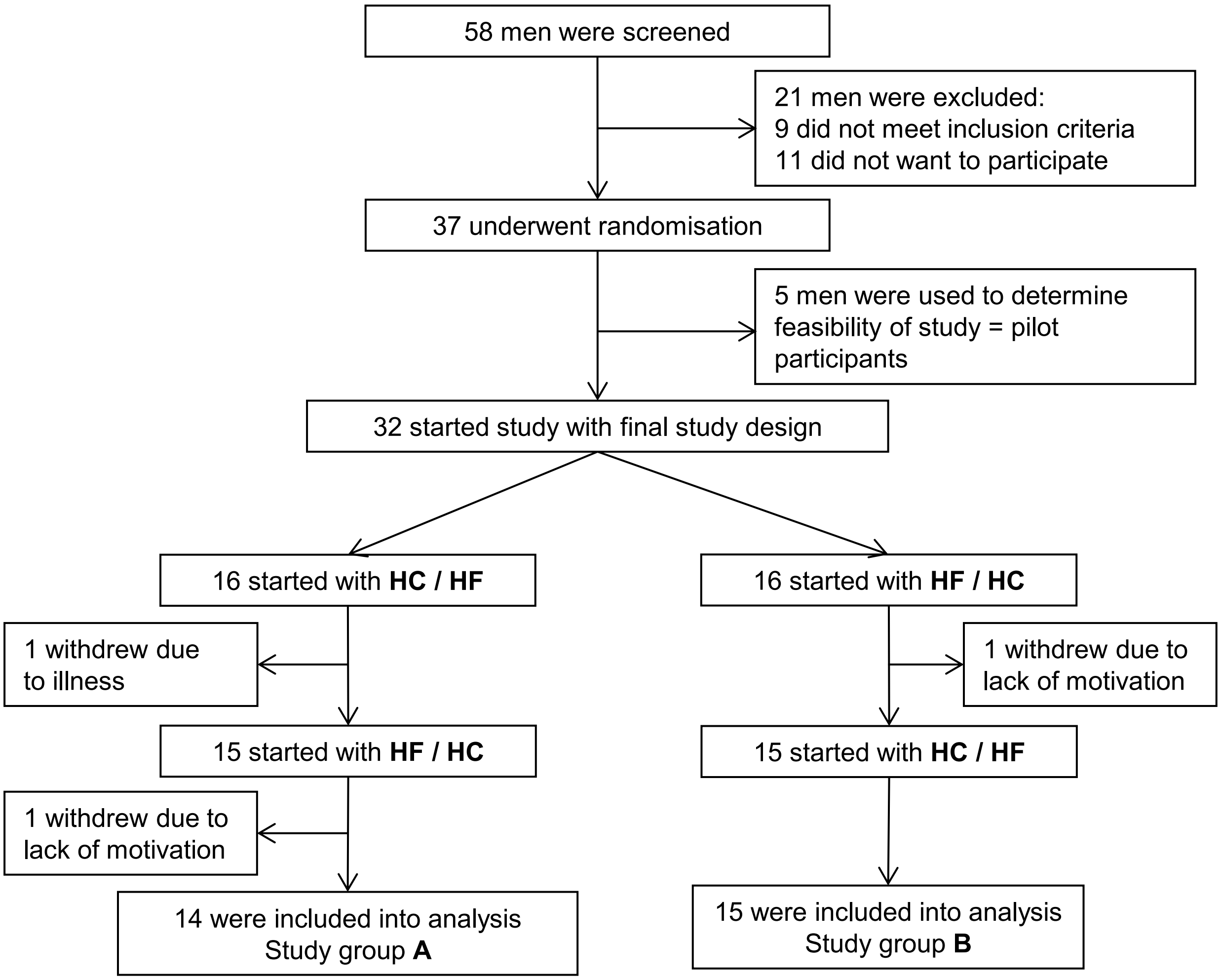

Figure S1. Flow of participants. HC/HF, isocaloric carbohydrate-rich diet in the morning and fat-rich diet in the evening; HF/HC, isocaloric fat-rich diet in the morning and carbohydrate-rich diet in the evening.

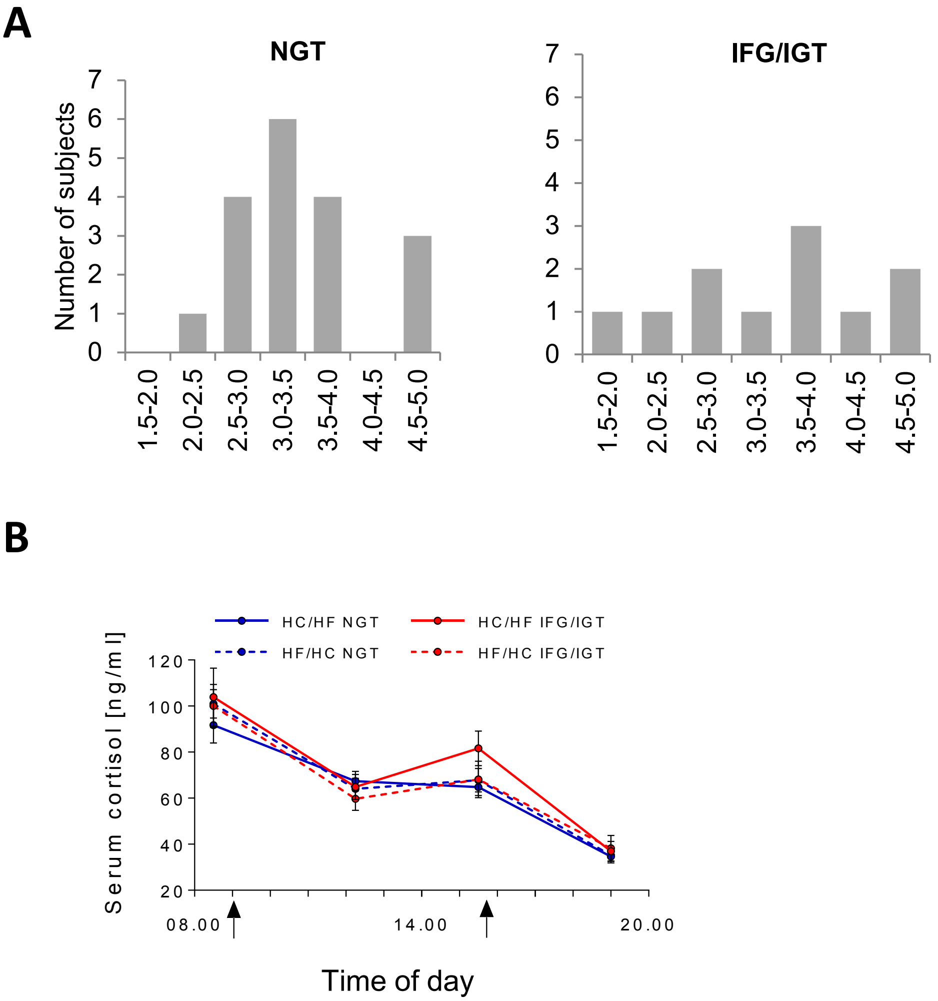

Figure S2. Circadian parameters of study participants.

(A) Chronotypes of NGT (n=18) and IFG/IGT (n=11) subjects determined using the MCTQ. Mid-sleep time-point on free days adjusted for individual average sleep need accumulated on work days (MSF-Sc) of all subjects is depicted. (B) Plasma cortisol levels measured at four time points (08.35 h, 12.15 h, 15.35 h and 19.00 h) during the investigation day.

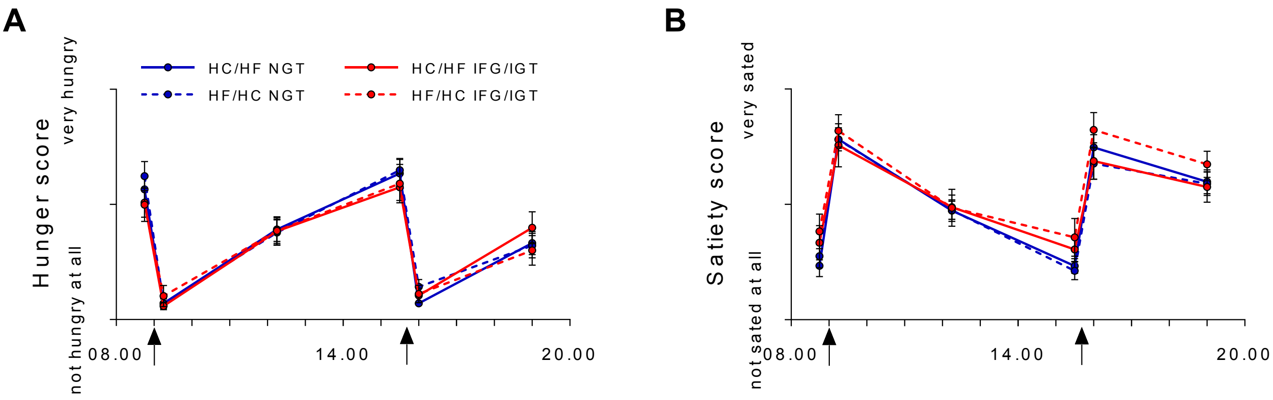

Figure S3. Hunger and satiety scores in response to the HC/HF diet and the HF/HC diet. Effects of the HC/HF diet (solid lines) and the HF/HC diet (dotted lines) on pre- and postprandial hunger (A) and satiety (B) scores in NGT subjects (blue) and IFG/IGT subjects (red). Arrow - test meal.

Table S1. Compliance during both dietary interventions

	HC/HF diet			HF/HC diet		
	06.00 – 22.00	06.00 – 13.30	16.30 – 22.00	06.00 – 22.00	06.00 – 13.30	16.30 – 22.00
Energy [KJ]	12073.4 ± 442.3	6197.1 ± 261.3§	5876.0 ± 212.0	11826.3 ± 415.1	6215.0 ± 252.9	5712.7 ± 207.5
CHO [EN %]	49.1 ± 0.7	64.8 ± 0.7	32.7 ± 1.2	48.7 ± 0.7	33.5 ± 0.9	65.0 ± 1.2
Fat [EN %]	36.3 ± 0.7	20.7 ± 0.5	52.4 ± 1.2	36.7 ± 0.8	51.4 ± 1.0	20.6 ± 1.0
Protein [EN %]	14.7 ± 0.2	14.4 ± 0.3	14.9 ± 0.3	14.6 ± 0.2	15.1 ± 0.3	14.4 ± 0.3
SFA [% from total fat]	42.8 ± 1.2	45.4 ± 1.1	41.8 ± 1.4	44.5 ± 1.0	43.9 ± 1.1	44.2 ± 1.4
Dietary GI	58.3 ± 0.5	59.4 ± 0.6	56.0 ± 0.9#	58.7 ± 0.5	59.9 ± 0.7	58.3 ± 0.5
Fibre [g]	35.5 ± 1.9	22.5 ± 1.6§	13.0 ± 0.7	32.9 ± 1.2	14.3 ± 0.6	18.6 ± 0.9
Starch [g]	174.3 ± 10.1	116.7 ± 8.5§	57.4 ± 3.3#	177.1 ± 6.5	79.7 ± 3.3	97.5 ± 4.8

Data are shown as mean ± SEM. §, $p < 0.05$, carbohydrate-rich diet in the morning (06.00 – 13.30) *versus* evening (16.30 – 22.00). #,$p < 0.05$, fat-rich diet in the morning *versus* evening). HC/HF, isocaloric carbohydrate-rich diet in the morning and fat-rich diet in the evening; HF/HC, isocaloric fat-rich diet in the morning and carbohydrate-rich diet in the evening; KJ, kilo joule; CHO, carbohydrates; EN %, energy percent; SFA, saturated fatty acids; GI, glycaemic index; GL, glycaemic laod.

Table S2. Example of a dietary plan for the HC/HF diet.

Food to be eaten		Alternative food
Morning block (breakfast + lunch) – to be eaten until 01.30 pm		
250 g	Orange Juice	107 g banana / 150 g apple / 174 g pear / 213 g mandarines / 141 g grapes / 266 g oranges / 174 g pineapple / 391 g strawberries / 260 g water melon / 112 g commercially available apple puree (tin)
50 g	Müsli	*Swiss style müsli*
150 g	Yoghurt (3.5% FDM)	159 g milk (3.5% FDM)
100 g	Low-fat curd	377 g semi-skimmed milk (1.5% fat) / 356 g yoghurt (1.5% fat) / 358 g buttermilk / 56 g ham / 55 g turkey breast
150 g	Apple	107 g banana / 174 g pear / 213 g mandarines / 141 g grapes / 266 g oranges / 174 g pineapple / 391 g strawberries / 260 g water melon / 112 g commercially available apple puree (tin) / 250 g fruit juice *(any kind)*
30 g	Sultanas	43 g dried apricots / 43 g dried plums / 33 g dried mango / 35 dried figs
120 g	Spaghetti (unboiled)	*equals to 300 g boiled spaghetti.* 109 g unboiled rice / 126 unboiled couscous / 126 g unboiled bulgur / 186 g bread / 104 g unboiled Chinese noodles
200 g	*Bertolli*® tomato sauce with basil	*any tomato sauce with similar composition*
10 g	Parmesan	
30 g	Milk chocolate	30 *Kitkat*® chocolate bar / 29 g *Sondey*® double choc cookies / 30 g *Snickers*® / 29 g *Bounty*® / 27 g *Twix*®
**Energy [kcal]**		**1400.8**
**CHO [EN %]**		**65.1**
**Fat [EN %]**		**19.9**
**Protein [EN %]**		**15.0**
Evening block (snack + dinner) – to be eaten between 04.30 and 10.00 pm		
200 g	Landliebe® Vanilla cream pudding	*Any kind of cream pudding with a similar composition*
40 g	Roasted peanuts	30 g walnuts / 32 g hazelnuts / 44 g roasted cashewnuts
135 g	Wheat roll	*Any kind of bread*
25 g	Butter	
45 g	Salami	32 g tea sauge spread / 47 g black pudding / 46 g calves liverwurst/ 54 g semi-hard cheese (45 % FDM) / 70 g Mozzarella / 46 g cream cheese (60 % FDM) / 49 g hummus
40 g	Ham	43 g corned beef / 40 g turkey breast / 42 g smoked pork chop / 72 g cottage cheese (20% FDM) / 30 g harz cheese / 72 g low fat curd
**Energy [kcal]**		**1402.3**
**CHO [EN %]**		**35.0**
**Fat [EN %]**		**50.4**
**Protein [EN %]**		**14.6**

Example of an individual dietary plan for the HC/HF diet adjusted for participant´s energy expenditure and food preferences. If possible 3-7 alternative food items were provided containing the same amount of the major macronutrient as the plan´s original food item. HF/HC, isocaloric fat-rich diet in the morning and carbohydrate-rich diet in the evening; Kcal, kilo calories; CHO, carbohydrates; EN %, energy percent; FDM, fat in dry matter.

Table S3: Example of a dietary plan for the HF/HC diet.

Morning block (breakfast + lunch) – to be eaten until 01.30 pm		
70 g	Wheat bread roll	*Any kind of bread*
10 g	Butter	
25 g	Nutella®	*Any kind of chocolate spread with similar composition*
90 g	Wheat bread roll	*Any kind of bread*
350 g	Tomatoes	455 g cucumber / 147 g peppers / 140 g carottes / 140 g beet root / 248 g kohlrabi / 464 g courgette / 413 g cellery / 420 g radish
120 g	Mozzarella	115 goat´s cheese / 105 g feta cheese / 82 g Gouda cheese
15 g	Olive oil	*Any kind of oil*
10 g	Vinegar	*Any kind of vinegar*
30 g	Roasted peanuts	22 g walnuts / 25 g hazelnuts / 33 g roasted cashewnuts / 126 g avocado / 113 g olives
**Energy [kcal]**		**1406.1**
**CHO [EN %]**		**34.9**
**Fat [EN %]**		**50.3**
**Protein [EN %]**		**14.8**

Evening block (snack + dinner) – to be eaten between 04.30 and 10.00 pm		
150 g	Apple	107 g banana / 174 g pear / 213 g mandarines / 141 g grapes/ 266 g oranges / 174 g pineapple / 391 g strawberries / 260 g water melon / 112 g commercially available apple puree (tin) / 250 g fruit juice *(any kind)*
107 g	Banana	150 g apple / 174 g pear / 213 g mandarines / 141 g grapes / 266 g oranges / 174 g pineapple / 391 g strawberries / 260 g water melon / 112 g commercially available apple puree (tin) / 250 g fruit juice *(any kind)*
54 g	Wine gums	59 g soft cake / 57 g lady finger / 56 g pretzel sticks / 390 g coke / 64 g marshmallow treat
130 g	Spaghetti (unboiled)	*Equals to 375 g boiled spaghetti.* 118 g unboiled rice / / 136 unboiled couscous / 136 g unboiled bulgur / 200 g bread / 112 g unboiled Chinese noodles
13 g	Olive oil	*Any kind of oil*
200 g	*Bertolli*® tomato sauce with basil	*Any tomato sauce with similar composition*
75 g	Ham	78 g corned beef / 73 g turkey breast / 77 g smoked pork chop / 74 g tuna / 134 g cottage cheese (20% FDM) / 134 g low fat curd / 91 g prawns / 506 g semi-skimmed milk / 482 g buttermilk
20 g	Parmesan	
300 g	Orange Juice (100%)	128 g banana / 180 g apple / 208 g pear / 256 g mandarines / 169 g grapes / 313 g oranges / 209 g pineapple / 469 g strawberries / 209 g water melon / 134 g commercially available apple puree (tin)
**Energy [kcal]**		**1400.8**
**CHO [EN %]**		**65.1**
**Fat [EN %]**		**19.9**
**Protein [EN %]**		**15.0**

Example of an individual dietary plan for the HF/HC diet adjusted for participant´s energy expenditure and food preferences. If possible 3-7 alternative food items were provided containing the same amount of the major macronutrient as the plan´s original food item. HF/HC, isocaloric fat-rich diet in the morning and carbohydrate-rich diet in the evening; Kcal, kilo calories; CHO, carbohydrates; EN %, energy percent; FDM, fat in dry matter.

Table S4. Composition of provided test meals.

	MTT-HC			MTT-HF		
	120 g	Wheat bread roll		119 g	Wheat bread roll	
	50 g	Wheat toast		17 g	Butter	
	12.5 g	Butter		19 g	Cheese (45 % FDM)	
	65 g	Strawberry jam		50.1 g	Philadelphia ®	
	16.7 g	Philadelphia®		150 g	Full-fat curd	
	110 g	Low-fat curd		250 g	Water	
	250 g	Water				
**Energy [kcal]**			**835.2**			**849.0**
**CHO [EN %]**			**64.8**			**35.3**
**Fat [EN %]**			**20.3**			**49.6**
**Protein [EN %]**			**14.8**			**15.1**
**SFA [% from total fat]**			**59.5**			**63.3**

MTT, meal tolerance test; HC, carbohydrate-rich; HF, fat-rich; FMD, fat in dry matter; CHO, carbohydrates; SFA, saturated fatty acids; kcal: kilo calories; EN %: energy percent
